# Genome-wide analysis of murine bone marrow-derived very small embryonic-like stem cells reveals that mitogenic growth factor signaling pathways play a crucial role in the quiescence and ageing of these cells

**DOI:** 10.3892/ijmm.2013.1389

**Published:** 2013-05-23

**Authors:** KATARZYNA MIERZEJEWSKA, JINBEOM HEO, JEONG WOOK KANG, HYUNSOOK KANG, JANINA RATAJCZAK, MARIUSZ Z. RATAJCZAK, MAGDA KUCIA, DONG-MYUNG SHIN

**Affiliations:** 1 Stem Cell Institute at the James Graham Brown Cancer Center, University of Louisville, Louisville, KY, USA; 2 Department of Medicine, Graduate School, University of Ulsan, Seoul, Republic of Korea

**Keywords:** very small embryonic-like stem cell, insulin/insulin-like growth factor signaling, gene set enrichment analysis, single-cell microarray

## Abstract

It has been postulated that the most primitive population of stem cells, Oct4^+^Sca-1^+^Lin^−^CD45^−^ very small embryonic-like stem cells (VSELs), differentiate into tissue-committed stem cells in adult mice. However, Oct4^+^ VSELs remain quiescent in adult tissues and do not form teratomas. In thi study, we report the characteristics of the VSEL transcriptome by gene set enrichment analysis employing a microarray database established from 20 murine bone marrow-derived, FACS-sorted VSELs in comparison with hematopoietic stem cells and embryonic stem cells. In the Oct4^+^ VSELs, we observed the upregulation of tissue-specific gene sets and a gene set encoding the complement-coagulation cascade. By contrast, in the VSELs, we observed the downregulation of genes involved in the UV radiation response, mRNA processing and mitogenic growth factor signaling [e.g., insulin-like growth factor-1 (IGF-1) and neurotrophic tyrosine kinase receptor A (TRKA), as well as the ERK and PI3K pathways]. Employing leading-edge subset analysis and real-time PCR assays, we observed that several genes, such as growth factor receptor-bound protein 2 (*GRB2),* son of sevenless homolog 1 *(SOS1),* SHC (Src homology 2 domain containing) transforming protein 1 *(SHC1),* mitogen-activated protein kinase kinase 1 *(MAP2K1),* v-akt murine thymoma viral oncogene homolog 3 *(AKT3), ELK1,* ribosomal protein S6 kinase, 90kDa, polypeptide 3 *(RPS6KA3),* glycogen synthase kinase 3*β (GSK3β)* and casein kinase 2, alpha 1 polypeptide (*CSNK2A1)*, which are involved in mitogenic growth factor signaling pathways, were commonly downregulated in the VSELs. Notably, this repression was reversed in the VSELs co-cultured over a C2C12 supportive cell-line, whereby they are induced to form VSEL-derived spheres (VSEL-DSs); thus, they are enriched, forming more differentiated stem cells. Therefore, we suggest that the repression of mitogenic growth factor signaling (e.g., through the IGF-1 receptor) may prevent uncontrolled Oct4^+^ VSEL proliferation and teratoma formation. Thus, restoring the responsiveness to mitogenic growth factors may be a crucial step in employing these cells in regenerative medicine.

## Introduction

Rapid progress in regenerative medicine has increased the demand for safe and therapeutically efficient sources of pluripotent stem cells (PSCs), which can differentiate into cells from all 3 germ layers (1). Typically, PSCs were established from embryonic tissues [e.g., embryonic stem cells (ESCs)] (2) and by the ectopic expression of reprogramming factors in terminally differentiated adult cells (e.g., inducible PSCs) (3). However, safety issues concerning inducible PSCs delays their application in clinical practice. Over the past few years, several attempts have been made to purify a population of PSCs from adult tissues. Potential PSCs in adult tissues have been described as i) mesenchymal stem cells (MSCs), ii) multipotent adult progenitor cells (MAPCs), iii) marrow-isolated adult multilineage inducible (MIAMI) cells, and iv) multipotent adult stem cells (MASCs) (4–7). It is conceivable that these cells, described by different investigators and given various names according to circumstances, may be closely related, overlapping populations of stem cells.

In a previous study, we demonstrated the presence of pluripotent Oct4^+^Sca-1^+^Lin^−^CD45^−^ very small embryonic-like stem cells (VSELs) in adult murine tissues (8). VSELs are slightly smaller than erythrocytes and express several markers of i) pluripotency [octamer-binding transcription factor 4 (*Oct4), Nanog, Sox2* and stage-specific embryonic antigen-1 (*SSEA-1)*], ii) the epiblast [gastrulation brain homeobox 2 (*Gbx2), fibroblast growth factor 5 (Fgf5)* and *Nodal*] and iii) epiblast-derived migratory primordial germ cells (PGCs) [*Stella, Blimp1* and PR domain containing 14 (*Prdm14*)] (9). The true expression of *Oct4, Nanog* and *Stella* in murine bone-marrow (BM)-derived VSELs has been confirmed by demonstrating the demethylated state of the DNA and enrichment analysis for transcriptionally active histone codes in the promoters of these genes (10). As we have previously demonstrated, VSELs can differentiate into cells from all 3 germ layers in *in vitro* culture conditions (8), and by employing several *in vivo* tissue regeneration animal models, we have proven that VSELs can be specified *in vivo* into mesenchymal stem cells (MSCs) (11), cardiomyocytes (12) and long-term engrafting hematopoietic stem cells (HSCs) (13). We hypothesized that these developmentally early stem cells are deposited during embryogenesis in adult tissues and reside there as a backup for monopotent tissue-committed stem cells (TCSCs), which rejuvenate specific organs.

We previously reported that these primitive BM-derived Oct4^+^ VSELs do not proliferate *in vitro* if cultured alone and do not form teratomas (8). The possible explanation for this finding is that VSEL proliferation is negatively regulated by the epigenetic reprogramming of certain imprinted genes [the insulin-like growth factor (*IGF)*-*2*-*H19* locus, *IGF-2R* and Ras protein-specific guanine nucleotide-releasing factor 1 (*Rasgrf1*)], which are involved in the insulin/IGF signaling (IIS) cascade (10). Specifically, in murine BM-derived VSELs, the paternally methylated imprints are erased at the *IGF-2-H19* and *Rasgrf1* loci and the maternally methylated imprints (e.g., at the *IGF-2R* locus) are hypermethylated. As a result, VSELs exhibit expression patterns characteristic of imprinted genes, which tends to attenuate responsiveness to the IIS pathway (10). Since the level of IGF-1 in blood plasma negatively correlates with longevity (14), we hypothesized that the genomic imprint-mediated repression of the IIS pathway may prevent the accelerated depletion of VSELs from adult tissues. This hypothesis has been supported by our recent studies on murine models of longevity (15–17). In particular, VSELs isolated from normal wild-type (wt) 2-year-old mice show increased DNA methylation of the differentially methylated regions (DMRs) at both the *IGF-2-H19* and *Rasgrf1* loci when compared with younger (2-month-old) mice (15). In addition, long-living plasma IGF-1-deficient Ames and Laron dwarf mice have significantly higher numbers of VSELs at the age of 2 than their wt littermates (17). By contrast, short-living bovine growth hormone (bGH)-overexpressing transgenic mice, with high levels of circulating IGF-1, display reduced numbers of VSELs (16). The changes in the numbers of VSELs in these animals correlated with the methylation of DMRs at the *IGF-2-H19* and *Rasgrf1* loci. As we previously reported, these loci were hypomethylated in long-living dwarf mice, but hypermethylated in short-living bGH-transgenic animals (17). Taken together, these findings suggest that the repression of IIS in VSELs in adult organs may have a beneficial effect on the life span of these cells.

In the present study, to gain the molecular insight that would allow us to modulate the quiescence of VSELs, we performed gene set enrichment analysis (GSEA) of a single-cell global transcriptome database, as reported in our previous study (18). In the Oct4^+^ VSELs, we observed the downregulation of genes involved in the UV response, mRNA processing and mitogenic growth factor signaling. Furthermore, we found that several genes, including growth factor receptor-bound protein 2 (*GRB2),* son of sevenless homolog *(SOS)1,* SHC (Src homology 2 domain containing) transforming protein 1 *(SHC1),* mitogen-activated protein kinase kinase 1 *(MAP2K1),* v-akt murine thymoma viral oncogene homolog 3 *(AKT3), ELK1,* ribosomal protein S6 kinase, 90kDa, polypeptide 3 *(RPS6KA3),* glycogen synthase kinase 3β *(GSK3β)* and casein kinase 2, alpha 1 polypeptide (*CSNK2A1)* modulate mitogenic growth factor signaling pathways and the proliferation of VSELs. Thus, we suggest that the repression of mitogenic growth factor pathways (e.g., those involved in IIS) contributes to the VSEL quiescent state.

## Materials and methods

### Isolation of VSELs and HSCs from murine BM

The present study was performed in accordance with the guidelines of the Animal Care and Use Committee of the University of Louisville, School of Medicine and the Guide for the Care and Use of Laboratory Animals (Department of Health and Human Services, publication no. NIH 86–23). Murine mononuclear cells (MNCs) were isolated from the BM of pathogen-free, 4-week-old female and male C57BL/6 mice (Jackson Laboratory). Isolated by flushing bones, BM cell suspensions were lysed in BD lysing buffer (BD Biosciences, San Jose, CA, USA) for 15 min at room temperature and washed twice in phosphate-buffered saline (PBS). VSELs (Sca-1^+^Lin^−^CD45^−^) and HSCs (Sca-1^+^Lin^−^CD45^+^) were isolated by multi-parameter, live-cell sorting (MoFlo), as described in our previous study (8).

### Formation of VSEL-derived spheres (VSEL-DSs) and ESC culture

VSEL-DSs were cultured over a C2C12 murine myoblast feeder layer as previously described (8), and cells isolated from VSEL-DSs on day 7 were employed in the present study. Murine ESC-D3 cells purchased from ATCC (Rockville, MD, USA) were grown in a 0.1% gelatin-coated dish under 100 IU/ml ESGRO (Millipore, Billerica, MA, USA), as previously described (10).

### FACS-sorted, 20-cell gene expression profiling

We employed a microarray database representing a cDNA library established from 20 cells of FACS-sorted VSELs, HSCs or trypsinized ESC-D3 cells. All the procedures for 20-cell cDNA library synthesis, microarrays and data processing were described in our previous study (18). The microarray datasets discussed in the present study have been deposited in the NCBI Gene Expression Omnibus (GEO, http://www.ncbi.nlm.nih.gov/geo) and are accessible through GEO Series accession no. GSE29281. Functional analysis of the 20-cell microarray database was performed using GSEA and leading-edge subset analysis (Broad Institute, Cambridge, MA, USA). For general global transcriptome comparison, GSEA based on a curated functional gene sets (C2) database was set with a permutation number of ‘1000’, ‘collapse dataset to gene symbols’ as ‘true’ and permutation type as ‘phenotype’. The C2 functional gene set collections were based on online pathway databases, publications in PubMed and knowledge of the domain. By default, gene sets were ordered by a normalized enrichment score (NES). A detailed list of gene sets is available upon request.

### Real-time reverse transcriptase (RT)-PCR (qRT-PCR)

Total RNA from the FACS-sorted cells (~20,000) was isolated using the RNeasy Mini kit (Qiagen, Inc., Valencia, CA, USA), with removal of genomic DNA using the DNA-free™ kit (Applied Biosystems, Foster City, CA, USA). mRNA (10 ng) was reverse-transcribed with TaqMan Reverse Transcription reagents (Applied Biosystems) according to the manufacturer’s instructions. Quantitative assessment of the expression of target genes from the regular or 20-cell cDNA library was performed by qRT-PCR using an ABI PRISM 7500 Sequence Detection system (Applied Biosystems) with SYBR-Green PCR Master Mix (Applied Biosystems). The threshold cycle (Ct), the cycle number at which the fluorescence of an amplified gene reaches a fixed threshold, was subsequently determined, and relative quantification of the expression level of target genes was performed with the 2^−ΔΔCt^ method, using the mRNA level of GAPDH as an endogenous control gene and that of bone marrow mononuclear cells (BMMNCs) as a calibrator. All the primers used in qRT-PCR are available upon request.

### Statistical analysis

All the data in qRT-PCR analyses were analyzed using the Student’s t-test or one-way ANOVA with the Bonferroni post-hoc test. We used the GraphPad Prism 5.0 program (GraphPad Software, Inc., La Jolla, CA, USA) and P-values <0.05 or <0.01 were considered to indicate statistically significant differences.

## Results

In a previous study, we performed transcriptome analysis of 20 FACS-sorted cells for murine BM-derived VSELs, HSCs and the embryonic stem cell-line, ESC-D3 (18). In the current study, to gain further insight into the VSEL transcriptome, we performed GSEA using these microarray databases. GSEA evaluates microarray data at the level of gene sets that share a common biological function, chromosomal location, or regulation (19). When we examined the enrichment of functional gene sets (C2) with default settings, 282 and 1,108 gene sets out of the 1,390 gene sets examined were upregulated and downregulated, respectively, in the VSELs compared with the HSCs and ESCs. At the same time, only a few gene sets were found as significant at a false discovery rate (FDR) ≤0.25, possibly due to the small number of samples. When we estimated the statistical significance at a nominal P-value <1%, 7 gene sets were significantly enriched in VSELs and 44 were enriched in the HSCs and ESC-D3 cells. At a nominal P-value <5%, 10 gene sets were significantly enriched in the VSELs and 120 were enriched in the HSCs and ESC-D3 cells.

**Figure 1:**
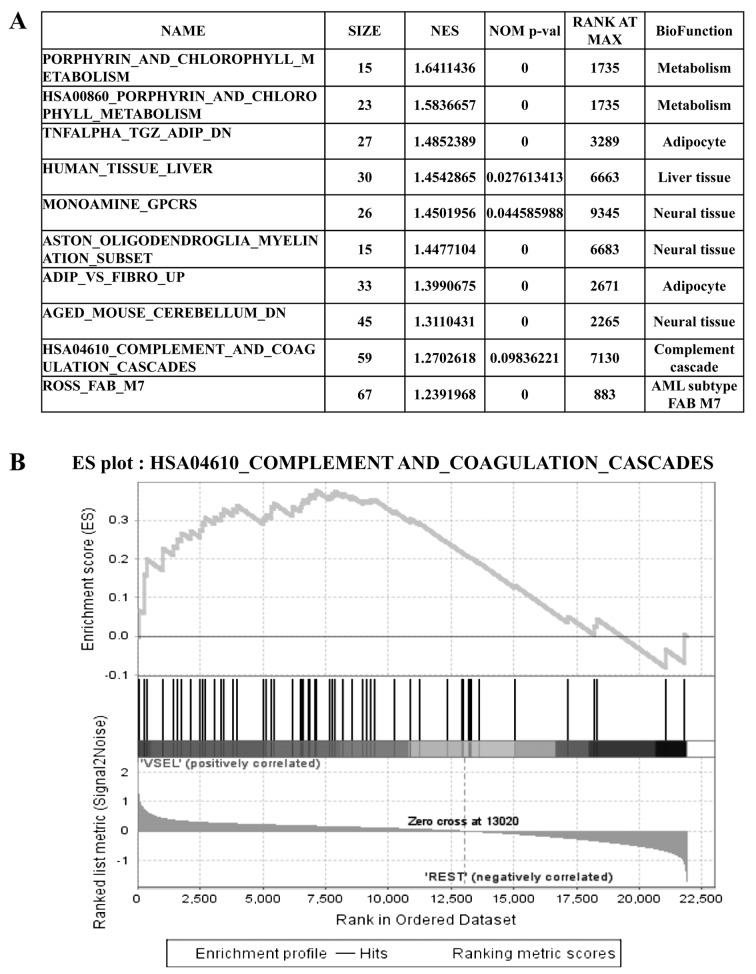
Gene sets that are upregulated in very small embryonic-like stem cells (VSELs). (A) Gene set list <5% of nominal P-value for the VSEL > rest of stem cells [hematopoietic stem cells (HSCs) and embryonic stem cell (ESC)-D3] comparison. Gene sets are listed according to the normalized enrichment score (NES). Dataset contains detailed gene set size, NES, nominal P-value (NOM P-value), ranked list position at which enrichment score reaches the maximum (Rank at MAX) and BioFunction. Several tissue lineage-specific gene sets (neural, adipose and liver tissues), as well as complement and coagulation cascade sets are included as VSEL upregulated genes. (B) Enrichment score (ES) plot for HSA HSA04610_COMPLEMENT AND_COAGULATION_CASCADES gene set.

GSEA provides the enrichment score (ES), which calculates the degree to which a gene set is overrepresented at the indicated cell phenotype. When we focused on the top-scoring 20 gene sets based on the NES, several tissue-specific genes characteristic of neural cells, adipocytes and liver tissues were highly enriched in the VSELs compared with the HSCs and ESC-D3 cells (Fig. 1). These results are in accordance with those from our previous study, showing that VSELs express several tissue-specific transcripts (8). However, to our knowledge, in this study, we report for the first time that the complement and coagulation cascade-related gene set was highly enriched in the VSELs compared with the HSCs and ESC-D3 cells (Fig. 1B). The complement cascade plays an important role in the mobilization of VSELs and HSCs in response to tissue injury (20,21). Thus, the enrichment of complement-coagulation cascade gene sets in VSELs supports their significant role in tissue and organ regeneration from injury.

**Figure 2:**
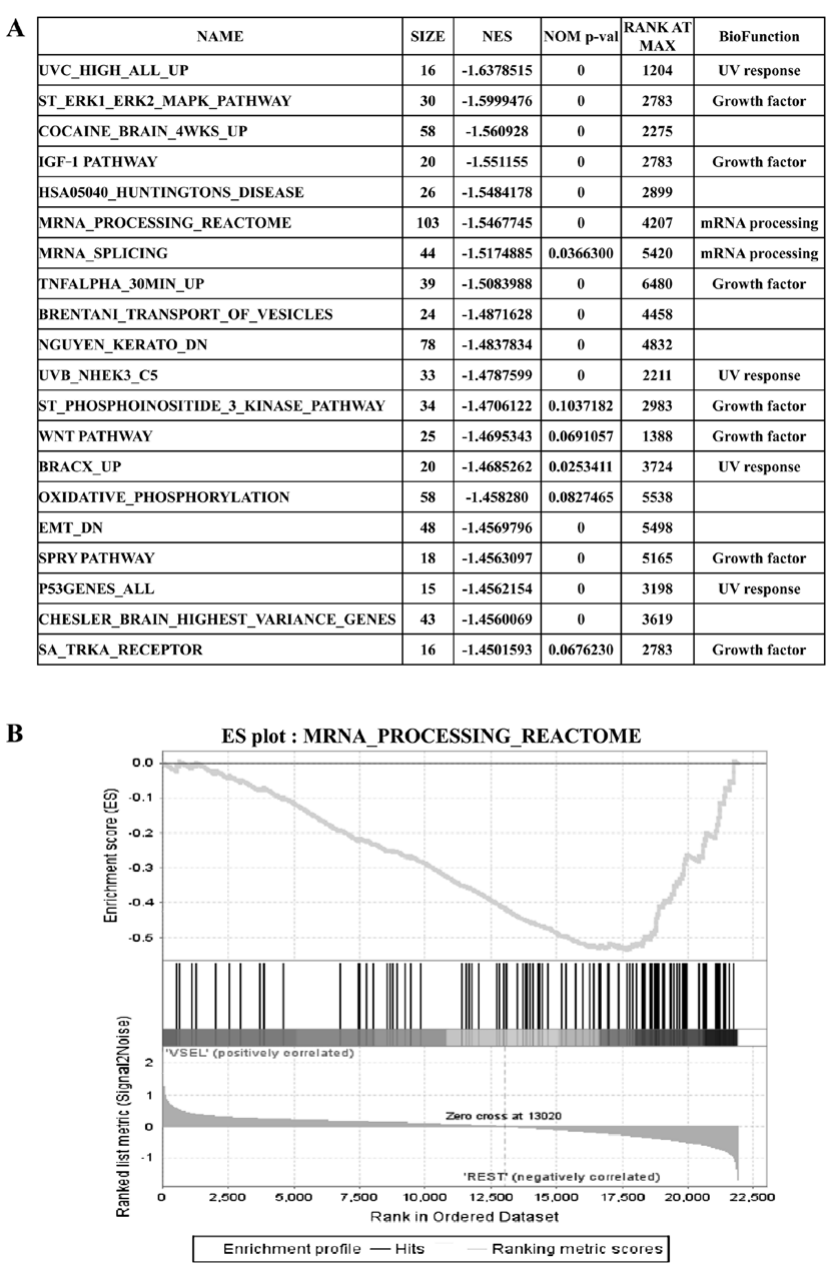
Gene sets that are downregulated in very small embryonic-like stem cells (VSELs). (A) The top-scoring 20 gene sets for the rest of stem cells [hematopoietic stem cells (HSCs) and embryonic stem cell (ESC)-D3] > VSEL comparison. Gene sets are listed according to the normalized enrichment score (NES). Gene sets for the UV response, mRNA processing, mitogen-activated and growth factor signaling pathways are downregulated in VSELs. (B) Enrichment score (ES) plot for MRNA_PROCESSING_REACTOME gene set.

Subsequently, when we explored the gene sets expressed at low levels in VSELs, we identified genes related to the UV response, mRNA processing, MAPK and PI3K signaling pathways, and mitogenic growth factor signal transduction (Fig. 2). Our previous demonstrated that murine VSELs are highly resistant to irradiation (13); this can be explained by the low expression levels of UV response-related gene sets (Fig. 2A). In addition, the low expression levels of genes involved in mRNA processing and cell proliferation (e.g., MAPK and PI3K pathways) (Fig. 2B) can explain why freshly isolated VSELs are quiescent and expand poorly in *ex vivo* cultures (10).

As mentioned above, VSELs remain quiescent due to epigenetic reprogramming of certain imprinted genes associated with IIS, such as *IGF-2-H19, Rasgrf1* and *IGF-2R*. As a result, VSELs have attenuated IIS and thus maintain their quiescence (10). By contrast, genes involved in IIS were highly upregulated in the HSCs and ESC-D3 cells (Fig. 2A). In addition, VSELs also expressed low levels of genes involved in the WNT and neurotrophic tyrosine kinase receptor A (TRKA) signaling pathways (Fig. 2A), which have been reported to be involved in stem cell proliferation (22–24).

**Figure 3:**
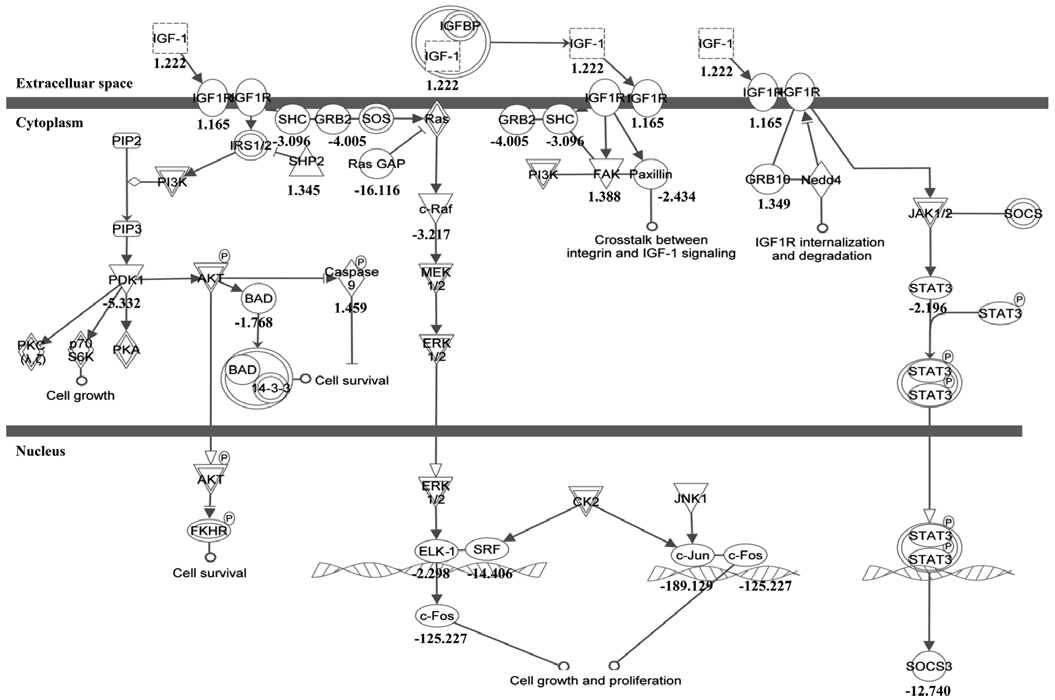
Overlay of the insulin-like growth factor-1 (IGF-1) signaling pathway with microarray data for comparison of very small embryonic-like stem cells (VSELs) with hematopoietic stem cells (HSCs). Relative expression is depicted as fold-change in gene expression. Fold-changes in gene expression between VSELs and HSCs are shown below the indicated genes. The canonical IGF-1 signaling pathway was built using ingenuity pathway analysis (IPA) software version 8.7.

Since VSELs have a significantly reduced expression of genes involved in IIS, we examined whether VSELs have a downregulated expression of the components of the IGF-1 signaling pathway. In order to address this issue, we employed ingenuity pathway analysis (IPA) software version 8.7 (Ingenuity Systems, Inc. Redwood City, CA, USA) and canonical pathway analysis to compare VSEL and HSC microarray data. VSELs displayed low expression levels of the components of the IGF-1 signaling cascade compared with HSCs (Fig. 3). Specifically, *FOS, JUN, JAK1, KRAS, SOS2,* serum response factor *(Srf),* suppressor of cytokine signaling 3 (*SOCS3*) and *SHC1* were significantly downregulated in the VSELs (Table I). Taken together, these results demonstrate that VSELs have a primitive gene expression pattern that tends to be resistant to IGF-1 signaling and other mitogenic growth factor pathways (e.g., ERK1/MAPK, TRKA, PI3K and WNT pathways).

**Figure 4:**
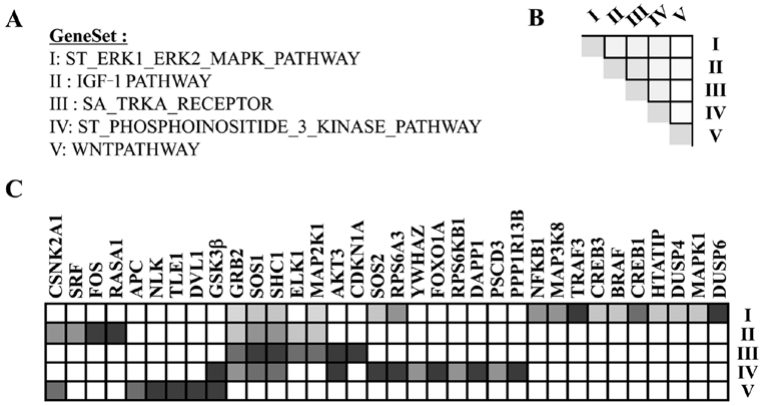
Leading-edge subset analysis for the mitogenic growth factor gene sets. (A) The list of gene sets used in leading-edge subset analysis. (B) Set-to-set analysis graph shows the overlap between subsets, which is represented by gray color intensity. A dark colored cell indicates that sets A and B have the same leading-edge genes and a white cell indicates that sets A and B have no common leading-edge genes. (C) Heatmap for the clustered genes in the leading-edge subsets. The level of downregulation in very small embryonic-like stem cells (VSELs) is depicted in dark gray color.

**Figure 5:**
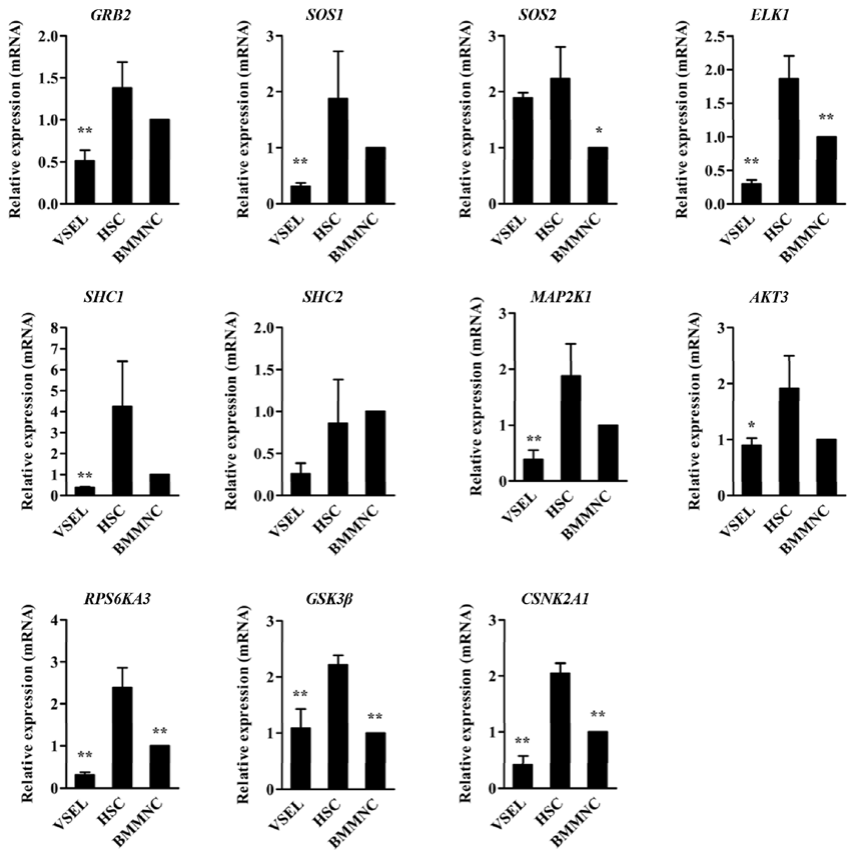
Expression of growth factor leading-edge genes in murine very small embryonic-like stem cells (VSELs). qRT-PCR analysis of genes specifically downregulated in leading-edge subset analysis (*GRB2, SOS1, SOS2, ELK1, SHC1, SHC2, MAP2K1, AKT3, RPS6kA3, GSK3β* and *CSNK2A1*) using the same cDNA library as in the microarray experiments. The expression level is represented as the fold difference relative to the value for bone marrow mononuclear cells (BMMNCs) and is presented as the mean ± SD of at least 3 independent experiments. ^*^P<0.05 and ^**^P<0.01 compared with hematopoietic stem cells (HSCs).

Subsequently, to identify VSEL-associated IGF-1- and mitogenic growth factor-related genes, we examined their leading-edge gene subsets, which represent the core members of high-scoring gene sets. Four gene sets, representing the ERK1/MAPK, IGF-1, TRKA and PI3K pathways (excluding WNT), showed a common biological function in VSELs (Fig. 4B). In a heatmap constructed to identify leading-edge genes, *GRB2, SOS1, SHC1, MAP2K1* and *AKT3* were common to these gene sets (Fig. 4C). To confirm our microarray results, we performed qRT-PCR analysis of the expression of these leading-edge genes. As expected, VSELs displayed a downregulated expression of the transcripts for *GRB2, SOS1, ELK1, SHC1, SHC2, MAP2K1, AKT3, RPS6KA3, GSK3β* and *CSNK2A1* (Fig. 5). By contrast, the expression of *SOS2* in the VSELs was only slightly downregulated.

**Figure 6:**
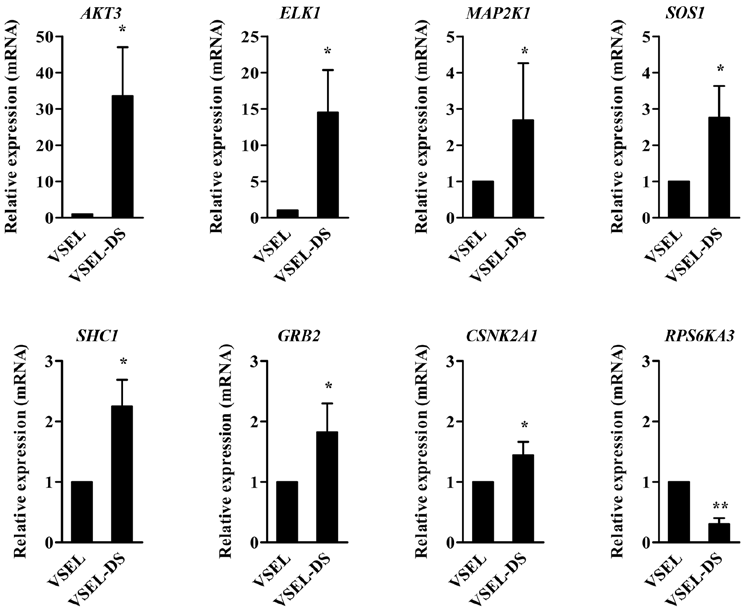
Expression of growth factor leading-edge genes is recovered during very small embryonic-like stem cell (VSEL)-derived sphere (VSEL-DS) formation. qRT-PCR analysis of the growth factor leading-edge genes (*AKT3, ELK1, MAP2K1, SOS1, SHC1, GRB2, CSNK2A1* and *RPS6KA3*) in freshly FACS-sorted VSELs and cells isolated from VSEL-DS. The expression level is represented as the fold difference relative to the value for VSELs and is presented as the mean ± SD of at least 3 independent experiments. ^*^P<0.05 and ^**^P<0.01 compared with VSELs. The graphs are ordered by the fold-change during VSEL-DS formation.

Previously, we reported that highly purified BM-derived Oct4^+^ VSELs do not proliferate *in vitro* if cultured alone. However in co-cultures with myoblastic C2C12 cells, VSELs form embryonic body (EB)-like structures described as VSEL-DSs that promote their proliferation capacity and differentiate into cells from all 3 germ layers (8). To address whether the leading-edge genes for the mitogenic growth factor gene sets are involved in modulating the proliferation of VSELs, we compared the expression of freshly isolated VSELs and cells isolated from 7-day-old VSEL-DSs. We observed that *AKT3* and *ELK1* were markedly upregulated during VSEL-DS formation (Fig. 6). Moreover, the expression of *MAP2K1, SOS1, SHC1, GRB2* and *CSNK2A1* increased only moderately in VSEL-DSs (Fig. 6). By contrast, the expression of *RPS6KA3* was decreased during VSEL-DS formation (Fig. 6). These data suggest that the leading-edge genes for mitogenic growth factor pathways that we identified in VSELs play an important role in maintaining the quiescence of primitive Oct4^+^ VSELs and preventing them from uncontrolled proliferation and forming teratomas.

## Discussion

In the present study, we demonstrated that murine BM-purified Oct4^+^ VSELs diplayed a downregulated expression of certain genes that encode mitogenic growth factor-related pathways, including the ERK1/MAPK, IGF-1, TRKA and PI3K pathways. The leading-edge genes controlling these pathways may be involved in modulating the proliferation of VSELs, which suggests that they prevent the ‘unleashed’ proliferation of these cells deposited during development in adult tissues.

GSEA of highly purified 20-cell transcriptomes demonstrated that VSELs had an increased expression of certain genes that regulate the complement and coagulation cascades (Fig. 1). The complement cascade plays an important role in the mobilization of stem cells (20). It has been reported that the most abundant product of complement cleavage/activation, which is the third complement component (C3) cleavage fragment (C3a) enhances the responsiveness of stem cells to stromal cell-derived factor-1 (SDF-1)/chemokine (C-X-C motif) ligand 12 (CXCL12) chemokine gradients (25,26). Since SDF-1-mediated CXCR4 signal transduction plays an important role in the trafficking of stem cells to damaged tissues, the enrichment of gene sets for the complement cascade in VSELs is explained by the need for their trafficking during tissue injury processes. Indeed, it has been reported that VSELs can be mobilized into peripheral blood during organ and tissue injury and as we hypothesized, circulate in an attempt to reach the damaged organs (27,28).

Of note, VSELs, as compared with HSCs and ESC-D3 cells, display a downregulated expression of genes involved in IIS (Fig. 2A), and thus express lower levels of several components of the IGF-1 signaling cascade (Fig. 3). In a previous study, we reported that VSELs remain in a quiescent state due to the reprogramming of selected genomic imprints, which results in the attenuation of their sensitivity to IIS (10). These changes lead to the downregulation of the expression of the *IGF-2, IGF-1R* and *Rasgrf1* genes, which promote IIS, and the upregulation of the non-signaling IGF-2-binding receptor encoded by *IGF-2R*. Taken together, VSELs employ the reprogramming of *IGF-2, Rasgrf1* and *IGF-2R* loci to protect themselves from autocrine/paracrine stimulation by insulin, IGF-1 and IGF-2. Our current microarray analysis demonstrated that VSELs additionally suppressed the expression of several genes involved in the intracellular IIS pathway (Fig. 2A). Specifically, leading-edge gene subset analysis revealed that several genes commonly involved in the mitogenic growth factor signaling pathways (e.g., ERK1/MAPK, TRKA and PI3K) pathways are repressed in VSELs (Fig. 4). Of note, the expression of these genes increased following the restoration of the VSEL proliferation capacity during VSEL-DS formation (Fig. 6). Therefore, our preliminary data identified new factors involved in maintaining the quiescence of VSELs; however, more complex studies employing, for example, RNA interference-mediated gene knockdown are required to verify these observations.

The quiescent state of VSELs provides numerous benefits for their normal physiological functions. First, we hypothesize that this is crucial for preventing these cells from uncontrolled proliferation and teratoma formation. Second, the premature depletion of VSELs may negatively affect their tissue rejuvenation potential. In support of this hypothesis, we have previously demonstrated that the pool of these cells decreases in adult murine tissues in an age-dependent manner (29,30) and that the epigenetic reprogramming of genes involved in IIS (*IGF-2-H19, Rasgrf1* and *IGF-2R* loci) prevents the premature depletion of VSELs (17). Moreover, de-repression of DNA methylation at these imprinted loci leads to the premature depletion of VSELs in adult tissues (17). Based on the current data, it is crucial to investigate whether the leading-edge genes identified in the present study, including *AKT3, ELK1, MAP2K1, SOS1, SHC1, GRB2* and *CSNK2A1* (Figs. 5 and 6), are involved in the ageing and tumorigenesis of VSELs.

In conclusion, in this study, we provide molecular evidence that murine Oct4^+^ VSELs have an upregulated expression of several other lineage-specific genes, as well as complement/coagulation pathways, but a downregulated expression of several genes involved in mRNA processing and growth factor/mitogen stimulation. We also identified several genes that are involved in the repression of the mitogenic growth factor pro-proliferation pathways. The modification of the expression of these genes is essential in order to further understand the involvement of VSELs in ageing and tumorigenesis, and may aid in the development of novel strategies for successfully employing these cells in clinical practice.

## Figures and Tables

**Table I:** Distinct expression of certain IGF-1 signaling components when comparing VSELs and HSCs.

Gene name	Gene symbol	Fold-change	P-value
Jun oncogene	*JUN*	−189.129	5.24E-07
FBJ murine osteosarcoma viral oncogene homolog	*FOS*	−125.22	7.12E-07
Janus kinase 1	*JAK1*	−34.637	2.56E-05
V-Ki-ras2 Kirsten rat sarcoma viral oncogene homolog	*KRAS*	−31.333	2.90E-05
Son of sevenless homolog 2 (*Drosophila*)	*SOS2*	−19.808	6.88E-04
Serum response factor	*SRF*	−14.406	−14.406
Suppressor of cytokine signaling 3	*SOCS3*	−12.740	1.01E-05
Growth factor receptor-bound protein 2	*GRB2*	−4.005	2.07E-04
SHC (Src homology 2 domain containing) transforming protein 1	*SHC1*	−3.096	1.60E-03
Mitogen-activated protein kinase kinase 1	*MAP2K1*	−2.483	6.15E-03
V-akt murine thymoma viral oncogene homolog 3	*AKT3*	−2.306	8.82E-02

Fold-change and P-values were calculated from the microarray data for the comparisin between VSELs and HSCs. The genes are ordered by the fold-changes in VSELs vs. HSCs. IGF-1, insulin-like growth factor-1; VSELs, very small embryonic-like stem cells; HSCs, hematopoietic stem cells.
